# Should nephrologists take a larger role in interventional nephrology, and should central line insertion remain a requirement of nephrology residency training? A debate

**DOI:** 10.1186/s40697-015-0045-x

**Published:** 2015-04-02

**Authors:** David C Mendelssohn

**Affiliations:** Humber River Hospital and University of Toronto, 200 Church St. Weston, Toronto, ON M9N 1 N8 Canada

## Abstract

The Canadian Society of Nephrology must soon provide input concerning the future of procedural training in nephrology. While at one time, the ability to insert a central venous catheter (CVC) was an essential skill required by all nephrologists, in 2014, nephrology training and practice has changed in fundamental ways such that it would be both unreasonable, and impractical, to maintain this requirement. Indeed, survey evidence suggests that many current trainees are not achieving this competency. Amongst the reasons that this requirement should be withdrawn include: 1) Not all trainees have the procedural skills to safely learn to insert CVC’s. 2) Most nephrologists in training and in practice are intellectually oriented, not procedurally oriented and are not seeking to perform lots of procedures. 3) In most practice settings, interventional radiologists and intensive care doctors perform dialysis line insertions using real time ultrasound guidance frequently, and offer timely, safer, and better service to patients. 4) Most trainees will not enter practice settings where CVC insertion ability is required. 5) Otherwise excellent future trainees may be denied a nephrology certificate of special competence only because they are unable to insert a CVC by the end of their fellowship. 6) Academic nephrology training programs that cannot provide adequate CVC insertion experience to fellows may lose their status as training centres. As a pragmatic way forward, Canadian nephrology training programs must encourage and offer only those nephrology trainees who have the ability and interest in procedural nephrology, a pathway through which they may be provided superb advanced training to become an expert. There is no longer a compelling reason to mandate this for all trainees.

## Abrégé

La Société Canadienne de Néphrologie doit bientôt donner son avis sur le futur de l’enseignement des compétences procédurales en néphrologie. Pouvoir insérer un cathéter veineux central (CVC) a longtemps été une compétence fondamentale pour tous les néphrologues; cependant, en 2014, la formation et la pratique de la néphrologie ne sont plus ce qu’elles étaient, et il serait à la fois déraisonnable et peu réaliste de vouloir conserver cette exigence.

En effet, les résultats de sondages laissent entrevoir que plusieurs résidents n’arrivent pas à combler cette exigence lors de leur formation. Parmi les raisons évoquées en faveur du retrait de cette exigence, on retrouve : 1) Tous les résidents ne détiennent pas les compétences procédurales nécessaires à l’insertion sécuritaire des CVC; 2) La majorité des néphrologues, qu’ils soient en formation ou en pratique, préfèrent le travail intellectuel aux procédures et ne cherchent pas à appliquer ce type de savoir-faire; 3) Dans la plupart des environnements de travail, les radiologistes interventionnels et les intensivistes insèrent les CVC de dialyse sous échographie de façon rapide et sécuritaire pour les patients; 4) La plupart des résidents ne travailleront pas dans un service qui exige du néphrologue qu’il insère les CVC; 5) Des résidents dont le parcours est excellent pourraient se voir refuser un certificat de compétence en néphrologie à la fin de leur résidence simplement parce qu’ils ne sont pas capables d’insérer un CVC; 6) Les programmes de formation universitaire en néphrologie qui ne peuvent pas fournir une exposition à la technique pratique considérée adéquate en matière d’insertion de CVC à ses résidents pourraient perdre leur statut de centre de formation.

Les programmes canadiens de formation en néphrologie doivent proposer une solution pragmatique à ce problème en continuant d’offrir l’enseignement des compétences procédurales aux résidents qui détiennent à la fois les habiletés et l’intérêt en la matière et en encourageant ceux qui sont intéressés par ces techniques à devenir des experts par le biais de formations plus poussées. Par contre, il n’existe aucune raison impérative d’exiger ceci de tous les résidents.

## Why is this article important?

This article is important because it challenges a traditional nephrology training requirement and argues that nephrology training should no longer require that every trainee learn to insert a central venous catheter for dialysis.

## Key messages

While at one time, the ability to insert a CVC was an essential skill required by all nephrologists, in 2014, nephrology training and practice has changed in fundamental ways such that it would be both unreasonable, and impractical, to maintain this requirement.

Trainees are in the best position to understand their passions, skills and desired career pathways, and, with guidance from nephrology program directors, can acquire the training they will need as they prepare to enter the job market. For some trainees and for some Canadian job opportunities, procedural training and the ability to insert CVC’s will be critical. However, in many circumstances these skills will not be required after completion of training, and therefore there should be no requirement for such training for all.

## Implications for future health policy

The Canadian Society of Nephrology has been asked to provide input to the Royal College of Physicians and Surgeons of Canada about this, and so it has potential public policy implications as nephrology training criteria are to be reassessed.

## Introduction

Acute or chronic hemodialysis (HD) requires arterial access to high blood flow rates from the patient to an extracorporeal circuit, and then a venous return of blood back to the patient. For chronic HD, Canadian and international guidelines recommend that an arterio-venous fistula (AVF) or graft (AVG) are preferred in suitable patients [1-4]. However, there are circumstances where such an access is not in place. When HD is required urgently for an acute indication or if chronic access is not in place, then timely insertion of a central venous catheter (CVC) is required. Indeed, swift insertion of a CVC leading to rapid initiation of HD, can be a life saving intervention.

There are many procedures that could be considered within the scope of nephrology practice. A partial list includes insertion of CVC, percutaneous renal biopsy, insertion of acute and chronic peritoneal dialysis catheters, and thrombolysis and/or angioplasty of AVF or AVG. This debate will focus on the one procedural skill that has traditionally been considered essential for every trainee to learn – the insertion of a CVC.

The Royal College of Physicians and Surgeons of Canada (RCPSC) sets the criteria and conditions whereby a nephrology trainee can acquire a certificate of special competence in nephrology (http://www.royalcollege.ca/cs/groups/public/documents/document/y2vk/mdaw/~edisp/tztest3rcpsced000917.pdf). Insertion of a central venous access remains a requirement for adult nephrology, as it has been since the birth of this subspecialty. Clearly at one time, this potentially life saving skill was considered to be fundamental and essential for any practicing nephrologist. However, the RCPSC is reconsidering this requirement and has asked the Canadian Society of Nephrology (CSN) for input. A similar discussion is occurring in the United States [5]. As part of its due diligence, CSN held a debate on November 12, 2014 in Philadelphia. This paper is based upon that presentation.

This topic is an emotional one, with deeply held beliefs. Since the debate cannot be based on a deep and rich scientific literature, it must be argued based mostly on personal experiences and opinions. For the reader, I feel it is important to portray where I have come from on this issue and how my career and my opinions have evolved.

I trained in the mid 1980’s and my most important mentor was Dr. Robert Uldall, who was the quintessential procedural nephrologist [6]. He taught me how to insert femoral and subclavian lines, and I became an expert in both procedures. Later, I learned how to perform ultrasound guided percutaneous kidney biopsies. The first twelve years of my career were as a full time university based nephrologist, and involved performing, teaching and publishing my experiences with procedures [7,8]. My career then led me to a very different, community based practice, where I have been for the past 13 years. It is important to note that I was never taught how to access the internal jugular vein, or how to insert a central venous catheter (CVC) using real time ultrasound guidance.

### Physician training and career paths

Let me state bluntly and up front that procedural nephrology can be challenging and rewarding, and should be promoted for young nephrologists who might thrive with procedures as a central part of their practice. However, my university based teaching experiences taught me a critical lesson, which is central to this debate. I can say with certainty that not all trainees have the ability and/or the interest in procedural nephrology. Indeed frankly, some trainees whom I tried to teach were so unskilled that patient safety was a real and serious concern. Upon reflection, procedural ability is highly variable between individuals, and this biological fact of life should be no surprise to anyone.

Broadly speaking, young physicians who are procedurally oriented choose surgical career paths, while those who are intellectually oriented take career paths in internal medicine and its subspecialties. Arguably, nephrology is the most intellectual of all subspecialties. It is highly unlikely that intellectually oriented interns and residents facing career choices that might include internal medicine and its subspecialties, would consider the opportunity to perform lots of procedures to be an important factor in promoting nephrology as their top career choice. It is common knowledge that the surgical personality is different from the medical one. Consider the well known description of orthopaedic surgeons as needing to be strong as an ox, and twice as smart [9]. No one would describe nephrologists that way! To assume that all nephrologists would thrive doing more procedures is naive.

### Procedures and community based nephrology practice

As a senior community based nephrologist, it is possible that I could be directly involved with a patient who requires an urgent CVC. However, in my environment, interventional radiology provides excellent and timely support for procedures, and as my first choice, I would ask them to perform the procedure. If a dangerous delay was encountered, then a direct personal appeal for help would almost always overcome the problem. These interventionalists are performing procedures all day every day, and are experts at ultrasound guided line insertions. And when a radiologist is not available, an intensive care physician can insert a dialysis line.

**Figure 1:**
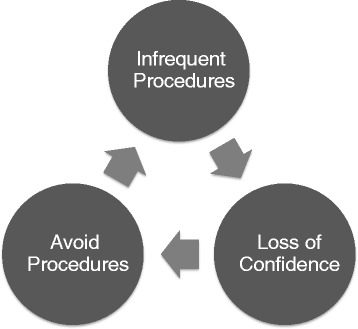
**A vicious cycle whereby performing procedures infrequently leads to loss of confidence, which leads to procedure avoidance.**

As a result of the availability of these experts who are more competent than me, I find myself caught in a vicious cycle (Figure 1). I perform procedures infrequently (once a year or less), and as a result I lose confidence in my ability to perform them well. As a result I avoid procedures by asking my colleagues to do them if they are available, which leads to even less opportunity to perform procedures.

This vicious cycle is reinforced by other factors intrinsic to procedures that intrude into an already busy schedule when I am alone caring for 30 to 50 inpatients or on call. Urgent procedures seem to never start on time, never end on time, and take up time that has not been pre-booked.

### Questions and answers

Let us consider the first part of the debate question, should nephrologists take a larger role in interventions? The way the question is framed is a problem for me. If we are talking about some nephrologists, then I am strongly supportive. If, on the other hand, we are promoting this for all nephrologists, then I am strongly opposed.

First of all, even within the nephrology subspecialty, there are many diverse career paths. Many of these will not require a nephrologist to perform procedures. Many young nephrology trainees are seeking skills to work in basic science labs and will practice in full time university settings where they will not perform procedures. Even in many community settings (like mine), procedural skills will not be required.

Secondly, the question needs to be considered in context. There are fundamental problems that exist within Canada that are impacting on trainees and the skill set they possess upon completion of their programs. Some Canadian academic teaching centres are reporting problems in providing an optimal procedural experience for their trainees. They report too few procedures to allow for competency to develop. Many nephrology faculty members are no longer sufficiently skilled to teach procedures to their own trainees. Passing off nephrology trainees to interventional radiology or ICU physicians for procedural training is not a solution either, since these disciplines have their own trainees who require an adequate volume of procedures.

Recent data exists that confirms this weakness of nephrology procedural training in Canada. Clark and colleagues in Ottawa have shown, in a national survey of trainees, that many do not become proficient in line insertion methodology [10].

Another troubling aspect of requiring all nephrologists to achieve competence in procedures relates to maintenance of that competency. If modern nephrology practice after completion of training leads only to rare or no opportunities to do procedures for the majority, then acquired skills will be lost. Once mandated to become competent, it seems like a contradiction to turn a blind eye to the maintenance of competency. If Canadian nephrology is already challenged with providing procedural training for many residents, then where will the political will and resources come from to provide avenues for all busy clinical nephrologists to maintain their skills?

Next let us consider what problems would be solved by maintaining the RCPSC requirements for training and what might be the consequences. Are the problems highlighted above causing negative patient care issues? There is a paucity of data in this regard, but I would submit that at least anecdotally, Canadian acute patients get timely access to high quality procedures, whether they are done by nephrologists or others. Furthermore, there is no evidence that nephrologists do these procedures faster than, or better than, others.

Even if one were to agree that every renal program should include procedural nephrology expertise, the next question becomes how many nephrologists are required to provide it? It seems to me that in most situations, one or two nephrologists would be the right number - more than that would be too many to maintain volumes and competency amongst them all. Indeed to extend this argument, if the right answer is that only a minority of nephrologists in any group would be the proceduralists, then why maintain CVC insertion as a requirement for all trainees?

Rigorous adherence to the current RCPSC requirement may also cause unintended consequences if rigidly enforced. It is not hard to conceive of a future trainee, excellent in all other competencies, who is denied the nephrology certificate of special competence solely because they cannot prove documentation of evidence of adequate training and certifiable skills in CVC insertion. It is equally possible to speculate that a training program would be put on probation, or even lose its training program status, if the RCPSC deemed it could not meet its CVC training obligations.

### Shaping the future of nephrology training and practice

Rather than insist that the status quo be defended at all costs, I suggest that CSN adapt its policies to create conditions that will lead to the evolution of a desired future landscape that fosters excellence in procedural clinical practice and patient care. What might that look like?

Certainly I would recommend that all nephrology trainees must continue to learn the fundamentals about CVC’s. Indications for specific locations, and short and long term complications might be part of a core curriculum. An expert panel of CSN might produce a document that outlines the learning objectives of such a curriculum.

CSN and Canadian nephrology training programs must encourage and offer nephrology trainees who have the ability and interest in procedural nephrology, a pathway whereby they may seek supplemental and advanced training to become an expert. There is no compelling reason to mandate this for all trainees at this time.

To achieve this desired model requires skilled and passionate teachers of procedural nephrology. It requires a sufficient volume of cases and an adequate case mix. It must include proficiency in real time ultrasound guidance for biopsies and line insertions, since this has become the standard of care [11]. Similarly, it must include simulation resources [12]. Simulation can augment and reinforce the teaching of techniques, but cannot replace actual clinical experiences.

In my opinion, it may not be possible for all Canadian academic centres to provide this high standard of procedural training. If not possible, then several university groups must develop and promote themselves as academic centres of excellence for procedural nephrology training. These centres of excellence would need to formalize relationships with other nephrology training programs, so that every Canadian nephrology trainee has the best opportunity to get advanced training, if desired. This might include electives during the R4 and/or R5 clinical training years, and supplemental 6 or 12 month fellowships after the years of compulsory training.

## Conclusions

This paper discusses several reasons why CVC insertion skill should no longer be mandated for all nephrology trainees (Table 1). These include: 1) Not all trainees have the procedural skills to safely learn to insert CVC’s. 2) Most nephrologists in training and in practice are intellectually oriented, not procedurally oriented, and are not seeking to perform lots of procedures. 3) In most practice settings, interventional radiologists and intensive care doctors perform dialysis line insertions using real time ultrasound guidance frequently, and offer timely, safer, and better service to patients. 4) Most trainees will not enter practice settings where CVC insertion ability is required. 5) Otherwise excellent future trainees may be denied a nephrology certificate of special competence only because they are unable to insert a CVC by the end of their fellowship. 6) Academic nephrology training programs that cannot provide adequate CVC insertion experience to fellows may lose their status as training centres.

**Table 1:** **Reasons not all trainees should learn to insert CVC’s**

1)	Not all have the skills to safely learn to do it
2)	Most are intellectually oriented, and are not seeking to perform lots of procedures
3)	Other physicians perform CVC insertions using real time ultrasound guidance frequently, and offer timely, safer, and better service to patients
4)	Most trainees will not enter practice settings where CVC insertion ability is required
5)	Otherwise excellent future trainees may be denied a nephrology certificate of special competence only because they are unable to insert a CVC
6)	Academic nephrology training programs that cannot provide adequate CVC insertion experience to fellows may lose their status as training centres

Trainees are in the best position to understand their passions, skills and desired career pathways, and, with guidance from nephrology program directors, can acquire the training they will need as they prepare to enter the job market. For some trainees and for some Canadian job opportunities, procedural training and the ability to insert CVC’s will be critical. However, in many circumstances these skills will not be required after completion of training, and therefore there should be no requirement for such training for all.

The CSN must soon provide input to the RCPSC about the future of training and practice in nephrology. While at one time, the ability to insert a CVC was an essential skill required by all nephrologists, in 2014, nephrology training and practice has changed in fundamental ways such that it would be both unreasonable, and impractical, to maintain this requirement.
